# Macular impairment in mitochondrial diseases: a potential biomarker of disease severity

**DOI:** 10.1038/s41598-020-65482-3

**Published:** 2020-05-22

**Authors:** Guido Primiano, Edoardo Abed, Giovanni Corbo, Angelo Maria Minnella, Serenella Servidei, Catello Vollono, Maria Cristina Savastano, Benedetto Falsini

**Affiliations:** 1 UOC Neurofisiopatologia, Fondazione Policlinico Universitario A. Gemelli IRCCS, Roma, Italy; 2 UOC Oftalmologia, Fondazione Policlinico Universitario A. Gemelli IRCCS, Roma, Italy; 3 Università Cattolica del Sacro Cuore, Roma, Italia

**Keywords:** Biomarkers, Prognostic markers

## Abstract

The high-energy demands of the retina are thought to contribute to its particular vulnerability to mitochondrial dysfunction. Photoreceptors are the cells with the higher oxygen consumption within the retina, and among these, the cones contain more mitochondria and have a higher energy demand than rods. A cohort of twenty-two patients with genetically-defined mitochondrial diseases (MDs) were enrolled to determine if the macula is functionally and anatomically impaired in these metabolic disorders. Visual acuity and fERG amplitude of patients with primary mitochondrial dysfunction were reduced compared to controls. Furthermore, SD-OCT layer segmentation showed a reduction of retinal and outer nuclear layer (ONL) volume in the macula of the patients. fERG amplitude showed a positive correlation with both ONL volume and thickness. A negative relationship was noted between fERG amplitude and disease severity assessed with Newcastle Mitochondrial Disease Adult Scale. In conclusion, MDs are associated with functional and anatomical alteration of macular cone system, characterized by its strong correlation with clinical disease severity suggesting a role as a potential biomarker of primary mitochondrial disorders.

## Introduction

There is growing evidence supporting an association between mitochondrial dysfunction and a number of retinal degenerations^1,2^. Several studies showed that mitochondrial genomic insufficiency may be linked to retinal aging^3^ and age-related macular degeneration (AMD) pathogenesis^4–6^. Indeed, the susceptibility of neural retina and retinal pigment epithelium (RPE) mitochondria to oxidative damage with aging appears to be a major factor in retinal degeneration.

Mitochondrial diseases are a heterogeneous group of diseases characterized by predominant neurologic and/or muscular involvements and associated with numerous ocular alterations including retinal degeneration, external ophthalmoplegia, and optic atrophy^7–9^.

Despite the tremendous progress in the molecular bases of mitochondrial diseases that, starting from 1989, lead to identification of mitochondrial DNA (mtDNA) defects and mutations in a growing number of nuclear genes that may produce or not mtDNA changes (multiple deletions or depletion)^10^, many aspects in the pathogenesis remain to be elucidated. The pathogenesis of retinal degeneration in mtDNA diseases may be related to the genetic abnormality and to the metabolic demand of this tissue^11^. The mitochondrion is the principal producer of ATP, and, at the same time, the primary source of reactive oxygen species (ROS). While in normal conditions the cell neutralizes ROS, in case of mtDNA mutation and/or insufficiency, the oxidative stress may exceed the capacity of cellular antioxidant mechanisms, leading to photoreceptors and RPE damage and apoptosis. Such mechanistic balance/unbalance could underlie the variety of retinal clinical pictures associated with mtDNA mutations, ranging from no clinical signs of retinal pathology to severe involvement.

The aims of this study were to determine if the macula, the retinal area with the highest energy demand, is altered in patients with mitochondrial disease (MD) and to investigate the chance to identify MD’s biomarkers in the eye.

To this purpose, both macular function and structure were quantitatively assessed by using focal electroretinogram and Spectral-Domain Optical Coherence Tomography (SD-OCT), respectively.

## Results

**Figure 1:**
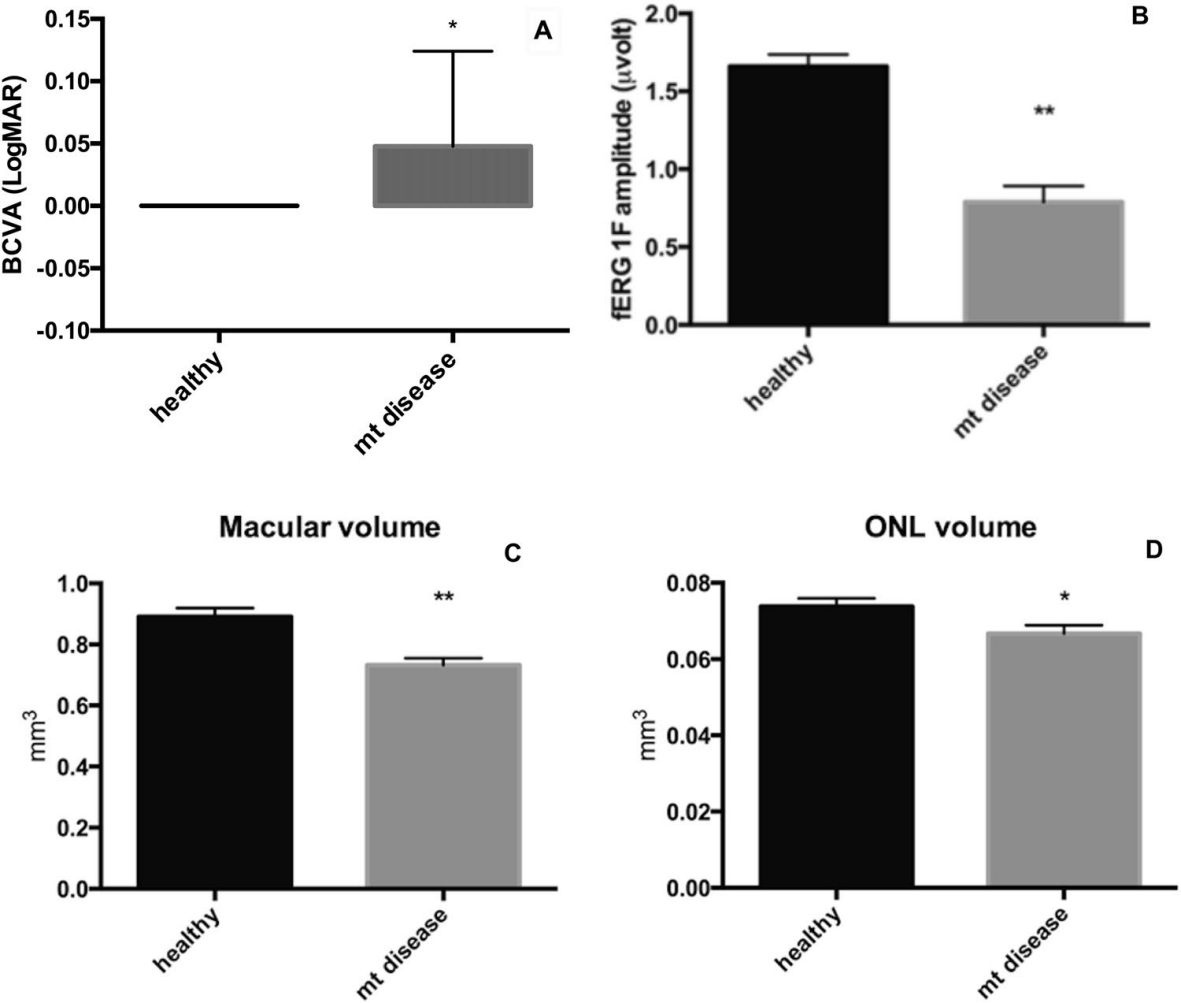
Box plot comparing mean ± SE BCVA (**A**) and fERG 1F amplitude (**B**) in patients with mitochondrial disease and control group *p < 0.01, **p < 0.0001; Box plot comparing mean ± SE macular (**C**) and ONL (**D**) volume in patients with mitochondrial diseases (mt disease) and control group *p < 0.05, **p < 0.001.

Demographics and clinical information of patients of the study group are reported in Table 1. All patients had normal kinetic visual fields and the maximum diameter of V4e isopter was 121 ± 13 degrees, which is comparable with the normative values previously reported by Niederhauser *et al*.^12^. Figure 1 shows a box plot where the BCVA and fERG 1F amplitude from the patients with MDs and control subjects are compared. Even if both BCVA and fERG fundamental harmonic were significantly reduced in the study group compared to controls the difference was more pronounced for 1F amplitude (BCVA: 10% reduction p < 0.01; fERG 1F: 52% reduction p < 0.0001, two-tailed unpaired t-test). Fundus examination did not show signs of any changes at posterior pole and at periphery in patients with mitochondrial disease. Similarly, Fundus autofluorescence (FAF), infrared (IR) imaging did not reveal any hypo or hypoautofluorescent lesion. Qualitative evaluation of SD-OCT scans by a skilled retinal specialist did not reveal significant alterations in correspondence of retinal and choroidal layers.

**Table 1:** Demographics and clinical data of patients with mitochondrial disease.

Patient	Age	Sex	Phenotype	Genotype	BCVA RE	BCVA LE	fERG RE (μV)	fERG LE (μV)	Goldmann RE V/4e isopter	Goldmann LE V/4e isopter	NMDAS
1	54	M	PEO	sDel mtDNA	0.22	0.15	0.08	0.15	110°	125°	34
2	44	M	PEO	mDel (POLG1, c.2864A>G)	0.10	0.22	0.18	1.01	140°	145°	22
3	42	F	MERRF	m.8356T>C	0.22	0.22	0.93	1.24	120°	132°	20
4	69	M	PEO	sDel mtDNA	0	0	0.94	1.51	122°	123°	13
5	74	F	PEO	sDel mtDNA	0.10	0	0.69	0.25	113°	110°	21
6	42	F	MERRF	m.8344A>G	0	0	1.08	0.87	130°	127°	17
7	60	M	MERRF	m.8344A>G	0	0	1.31	1.43	128°	120°	23
8	42	M	MNGIE	mDel (c.925C > G exon 7 and c.1231_1243del exon 9 TYMP)	0	0	0.29	0.15	105°	110°	25
9	62	F	PEO	mDel mtDNA	0	0	0.46	0.22	133°	141°	20
10	50	F	PEO	mDel mtDNA	0	0	1.57	1.74	150°	150°	9
11	44	F	PEO	sDel mtDNA	0	0	0.73	0.85	135°	140°	18
12	75	M	PEO	sDel mtDNA	0	0	1.85	1.33	120°	115°	15
13	31	F	PEO	sDel mtDNA	0.15	0.15	0.53	1.27	105°	97°	22
14	52	M	PEO	mDel mtDNA	0	0	0.64	0.76	130°	138°	17
15	63	F	PEO	mDel mtDNA	0.22	0.15	0.29	0.40	122°	105°	25
16	22	M	MNGIE	mDel (c.977G>A exon 8 and c.215-12_222del exon 3 TYMP)	0	0	1.17	1.24	112°	110°	17
17	64	F	MERRF	m.8344A>G	0.05	0.05	0.21	0.17	105°	100°	26
18	25	F	PEO	m.3243A>G	0.05	0.05	0.65	0.35	115°	120°	25
19	44	M	MERRF	m.8344A>G	0	0	1.25	1.29	130°	112°	8
20	41	M	MNGIE	mDel (c.925C > G exon 7 and c.1231_1243del exon 9 TYMP)	0	0	0.18	0.43	123°	131°	22
21	52	F	PEO	mDel (POLG1, c.1943C > G and c.2243 G > C)	0	0	0.11	0.45	112°	114°	24
22	51	F	MELAS	m.3243A>G	0	0	1.23	1.26	124°	118°	21

(NMDAS: Newcastle Mitochondrial Disease Adult Scale).

**Figure 2:**
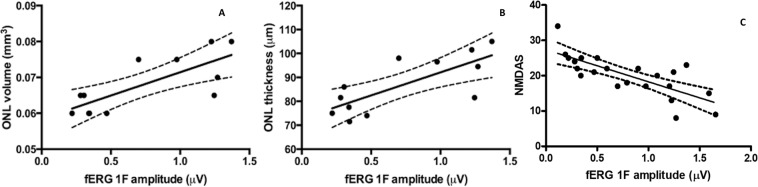
Scatterplot showing the positive correlation between fERG 1F amplitude and ONL volume (**A**) and thickness (**B**); scatterplot showing the inverse correlation between fERG 1F amplitude and Newcastle scale score in patients with mitochondrial disease (mt disease) (**C**).

In patients with mitochondrial diseases, segmentation analysis of SD-OCT scans showed a significant reduction of macular volume (0.89 ± 0.03 and 0.73 ± 0.02 p = 0.0002) and ONL volume (0.074 ± 0.002 and 0.067 ± 0.002 p = 0.03) compared with controls (Fig. 2) while no significant differences were noted in the volume of other retinal layers. No statistically significant differences were found in the thickness of any retinal layer even if the thickness ONL was slightly lower in patients with mitochondrial disease (93.3 ± 2.9 and 84.8 ± 3.6 p = 0.08).

Results of the comparison of retinal layer thickness and volume are summarized in Table 2.

**Table 2:** Comparison of thickness and volume of the entire retina and of individual retinal layers between healthy subjects and patients with mitochondrial disease (mean ± SD).

	RT	RNFL	GCL	IPL	INL	OPL	ONL	IRL	PR	RPE
**Retinal layers thickness**										
Mitochondrial disease	304 ± 6	13.3 ± 0.9	19.5 ± 3.7	23.7 ± 2.1	23.3 ± 2.7	25.9 ± 1.3	84.8 ± 3.6	190.3 ± 8.7	88.2 ± 1.2	16.4 ± 3.8
Controls	313 ± 4	13.4 ± 0.3	18.3 ± 1.5	21.3 ± 0.9	21.1 ± 1.4	25.5 ± 1.1	93.3 ± 2.9	191.1 ± 4.5	87.3 ± 0.9	17.8 ± 3.2
P	0.21	0.96	0.77	0.32	0.42	0.82	0.08	0.94	0.57	0.33
**Retinal layers volume**										
Mitochondrial disease	0.73 ± 0.02	0.010 ± 0.0	0.016 ± 0.002	0.019 ± 0.002	0.018 ± 0.002	0.019 ± 0.001	0.067 ± 0.002	0.14 ± 0.004	0.068 ± 0011	0.014 ± 0.002
Controls	0.89 ± 0.03	0.011 ± 0.0	0.013 ± 0.001	0.018 ± 0.001	0.018 ± 0.001	0.020 ± 0.0	0.074 ± 0.002	0.15 ± 0.013	0.069 ± 0.001	0.015 ± 0.002
P	0.0002	0.34	0.40	0.53	0.96	0.34	0.03	0.30	0.52	0.22

Linear regression with Pearson’s coefficient found a significant positive correlation between fERG 1F amplitude and both ONL volume and thickness (r2 = 0.57,p = 0.004 and r2 = 0.56,p = 0.005 respectively) (Fig. 2A,B). Conversely, an inverse relationship was noted between fERG 1F amplitude and Newcastle Mitochondrial Disease Adult Scale (NMDAS) (r2 = 0.55, p < 0.0001) as showed in the scatterplot (Fig. 2C). No significant correlation was found between fERG amplitudes and age of patients (r2 = 0.002, p = 0.96) or between BCVA and other clinical parameters.

## Discussion

The purposes of this study were to assess macular structure and function in patients with MDs and identify a potential eye biomarker for these genetic diseases. In our study cohort, both visual acuity and fERG first harmonic were significantly reduced compared to age-matched controls. This finding is consistent with previous experimental studies in mouse models of mitochondrial disease knockout of the SOD2 gene showing a progressive decline of retinal function^13^. As already demonstrated, fERG’s 1F is mainly generated by cone photoreceptors and their bipolar cells^14^, thus indicating a dysfunction of macular cones and bipolar cells in patients affected by mitochondrial dysfunction. It should be noted that cone system function impairment might be caused by a number of conditions associated with mitochondrial diseases such as diabetes^15^ and optic atrophy^16^. However, none of the patients enrolled had diabetes and the presence of optic atrophy was excluded, in all subjects, by visual field testing and fundus examination.

In ONL foveal region the body and nuclei of cone photoreceptors, key elements in ocular function, are anatomically placed. Interestingly, the segmentation analysis showed a significant reduction of macular volume and ONL volume in a subset of patients with mtDNA diseases compared with healthy controls while no significant differences were noted in the thickness and volume of the remaining retinal layers. This data demonstrates that mitochondrial disease causes a selective damage of macular photoreceptor cells with a preservation of other retinal layers and without significant pathological findings in the IR and FAF imaging suggesting that cone photoreceptor were mainly involved during the early phase in these patients.

The increased susceptibility of macular cones to oxidative damage may be related to the physiology of these cells. Photoreceptors are the cells with the higher oxygen consumption within the retina^17,18^. In experimental studies on rodents, Yu *et al*. demonstrated that the whole inner retina consumes approximately the 17% of the oxygen burned by photoreceptors^19^. Among photoreceptors, cones contain more mitochondria and have a higher energy demand than rods^20^.

Furthermore, photoreceptors cells have a reduced mitochondrial reserve capacity. A recent study by Kooragayala and colleagues^21^ showed that in photoreceptors the addition of uncoupling proteins cause an increase of only 25% of oxygen consumption compared with the 2–3 fold increase seen in other neural cells^22^.

The reduction of mitochondrial functional reserve causes a shift of ATP production from oxidative phosphorylation toward glycolysis and is linearly correlated with photoreceptors apoptosis^11^.

It is tempting to speculate that cone photoreceptors, the retinal cells with the greater oxygen consumption and characterized by a low mitochondrial reserve capacity may be more vulnerable to the mitochondrial dysfunction caused by mtDNA mutations.

A significant positive correlation was found between fERG’s 1F amplitude and both ONL thickness and volume, suggesting that there is a tight relationship between functional impairment and structural alterations of macular cones.

Interestingly, linear regression analysis showed a significant negative correlation between fERG fundamental harmonic amplitude and disease severity assessed with Newscastle Mitochondrial Disease Adult Scale. This finding suggests that, in mitochondrial encephalomyopathies, the systemic involvement and macular cone dysfunction share a common pathogenetic mechanism.

No correlation was noted between the amplitude of fERG first harmonic and increasing age. The relationship between mitochondrial damage and retinal aging and degeneration has been documented by a large number of studies. The RPE in elderly and patients with AMD is characterized by an accumulation of mtDNA mutations, an increase of mitochondrial heteroplasmy and a reduction of mtDNA repair capability^23^. Furthermore, it has been shown that specific mitochondrial haplogroups are associated with increased risk of developing AMD^24^. Hence, the lack of correlation between the decline of macular function and increasing age may be apparently surprising. However, it should be noted that, even if mitochondrial damage plays role in the development of both AMD and mitochondrial encephalomyopathies, the pathogenesis of these diseases is quite different. While MDs are fundamentally caused by defects in mitochondrial oxidative phosphorylation (OXPHOS), AMD has a multifactorial pathogenesis that results from the combination of different factors such as complement activation, inflammation and oxidative stress^25^. This discrepancy is further demonstrated by a post hoc analysis on AREDS study data showing that mitochondrial diseases are only rarely associated with age-related maculopathy^26^.

In conclusion, the present study demonstrates an involvement of macular function and structure in patients with primary mitochondrial dysfunction. The strong positive correlation between fERG’s 1F amplitude and NMDAS score makes the functional retina examination a reproducible and non-invasive investigation with a potential role as a biomarker of disease severity in MDs. These results are particularly relevant because of the need to have a reliable biomarker available for the forthcoming clinical trials in these genetic disorders. Further longitudinal studies should be able to establish if the diagnostic methods used in our study may provide a novel clinical biomarker of mitochondrial disease based on macular structure and function.

## Methods

### Patients

Twenty-two patients (10 males and 12 females, mean age 50.1 ± 14.4 years) affected by different MDs were included in this study in this retrospective, observational case series.

In each patient, the diagnosis was confirmed by mitochondrial DNA analysis and muscle biopsy. Our cohort included 13 patients affected by progressive external ophthalmoplegia (PEO) (6 single mtDNA deletion, 6  multiple mtDNA deletions and one with missense mutation), five patients with myoclonus epilepsy and ragged red fibers (MERRF) associated with m.8344A > G or m.8356T>C mutations, three patients with TYMP mutations and mitochondrial neurogastrointestinal encephalopathy (MNGIE) and one patient with mitochondrial encephalopathy lactic acidosis and stroke-like episodes (MELAS) and m.3243A > G mutation. All subjects were assessed using the NMDAS, a semiquantitative rating scale used to monitor a patient’s disease status in relation to other organ systems commonly involved in mitochondrial diseases^27^. Thirteen age-matched healthy patients were also enrolled and served as controls. Patients with diabetes, corneal opacities, cataract, glaucoma, optic nerve atrophy or any coexistent disease that was likely to limit visual function were excluded. Demographic, molecular and clinical characteristics of study patients are summarized in Table 1. Informed consent was obtained from patients and controls. The study was approved by the Ethics Committee of the Università Cattolica del Sacro Cuore in Rome and followed the tenets of the Declaration of Helsinki.

### Ophthalmological examinations

Each patient underwent a full ophthalmologic examination including detailed family history, anterior segment biomicroscopy, BCVA measurement with Snellen chart, indirect ophthalmoscopy, retinography, intraocular pressure measurement, Goldmann visual field examination and focal electroretinogram (fERG) recording. Kinetic visual fields were measured to the V4e white test light of the Goldmann perimeter against the standard background of 31.5 apostilbs. Fields were digitized and converted to areas and equivalent diameters. Fundus autofluorescence (FAF), infrared (IR) and SD-OCT imaging was performed in a subset of 12 patients.

### Electrophysiology

Focal electroretinograms were recorded from the central 18 degrees region using a uniform red field superimposed on an equiluminant steady adapting background, used to minimize stray-light modulation^28–30^. The stimulus was generated by a circular array of eight red LEDs (λ maximum, 660 nm; mean luminance, 93 cd/m^2^) presented on the rear of a Ganzfeld bowl (white-adapting background). A diffusing filter in front of the LED array made it appear as a circle of uniform red light. fERGs were recorded in response to the sinusoidal 95% luminance modulation of the central red field. Flickering frequency was 41 Hz. Patients fixated monocularly at a 0.25° central fixation mark, under the constant monitoring of an external observer. Before recording, pupils were pharmacologically (1% tropicamide and 2.5% phenylephrine hydrochloride) dilated to a diameter ≥ 8 mm and all subjects underwent a preadaptation period of 20 minutes to the stimulus mean luminance. fERGs were recorded by an Ag-AgCl electrode taped on the skin over the lower eyelid. A similar electrode, placed over the eyelid of the contralateral patched eye, was used as reference (inter-ocular recording). fERG signals were amplified (100,000-fold), bandpass filtered between 1 and 100 Hz (6 dB/oct), and averaged (12-bit resolution, 2-kHz sampling rate, 200–600 repetitions in 2–6 blocks). Off-line discrete Fourier analysis quantified the amplitude and phase lag of the first harmonic (1F) at 41 Hz.

### Fundus autofluorescence/infrared/optical coherence tomography

**Figure 3:**
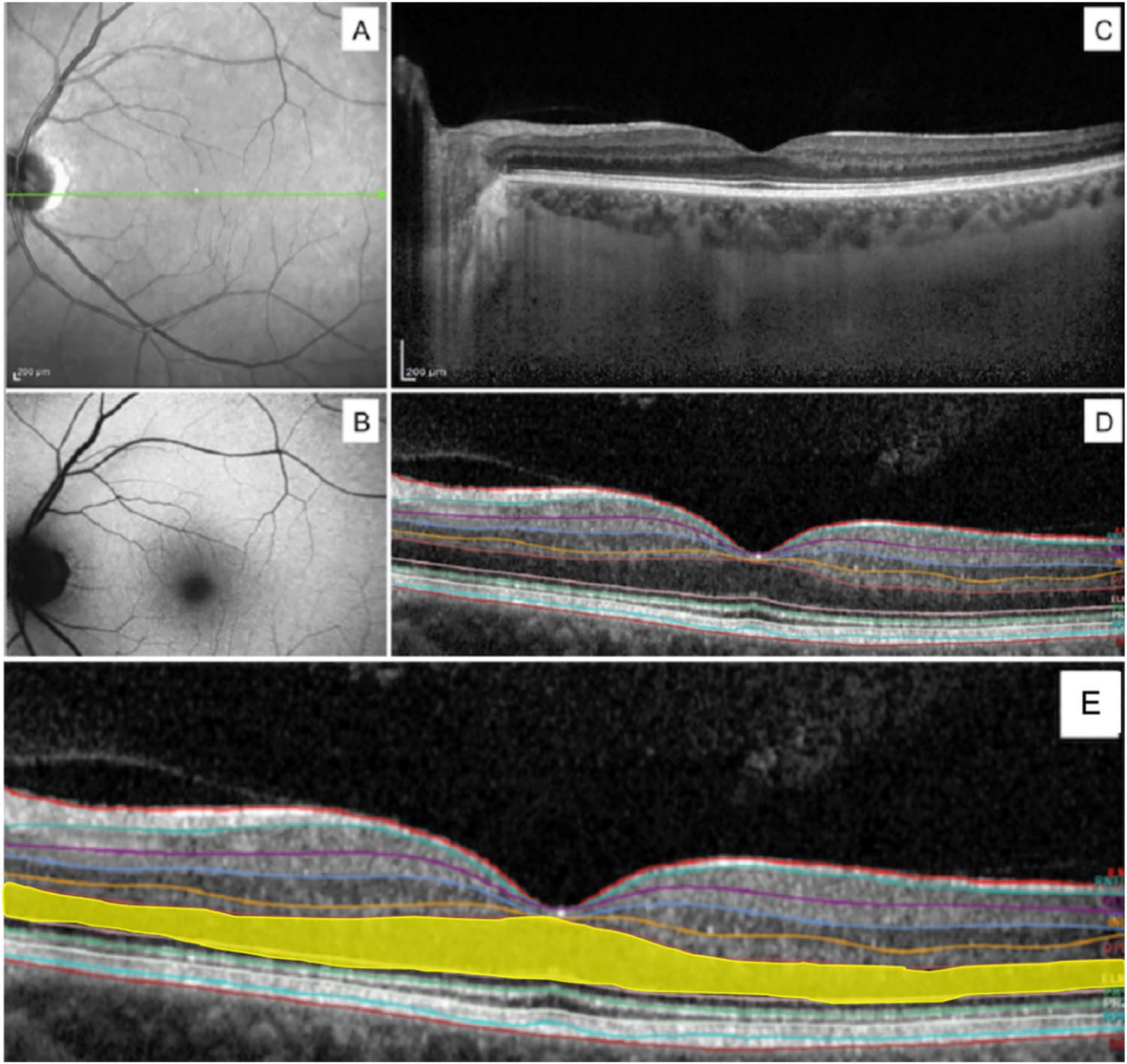
Representative example of the left eye of a patient with MELAS. Infrared (**A**), Autofluorescence (**B**), SD-OCT B-scan centered at the fovea with EDI mode (**C**); automated segmentation details of Spectralis (**D**). ONL highlighted in yellow for better visualization; IR and AF did not show changes; full macular volume and ONL volume decreased in SD-OCT analysis for eye’s MELAS patient (**E**). Inner limiting membrane (ILM), retinal nervous fiber layer (RNFL), ganglion cell layer (GCL), inner plexiform layer (IPL), inner nuclear layer (INL), outer plexiform layer (OPL), external limiting membrane (ELM), photoreceptor inner layer (PR1), photoreceptor outer layer (PR2), retinal pigment epithelium (RPE), Bruchs membrane (BM).

FAF, IR and SD-OCT imaging were performed by using Heidelberg Spectralis® (Heidelberg Engineering, Heidelberg, Germany) after fundus examination (Fig. 3). The SD-OCT examination protocol consisted of 19 to 25 horizontal b-scan lines centered at the fovea and covering the central 30° of the retina. Horizontal b-scans were acquired with the enhanced depth imaging (EDI) technique and using the automatic real-time mode (ART), averaging 18 frames per image. Segmentation analysis was automatically performed by Heidelberg Eye Explorer Software (Heidelberg Engineering, Heidelberg, Germany) which recognizes nine distinct retinal layers including retinal nerve fibre layer (RNFL), ganglion cell layer (GCL), inner plexiform layer (IPL), inner nuclear layer (INL), outer plexiform layer (OPL), outer nuclear layer (ONL), retinal pigment epithelium (RPE), inner retinal layers (IRL) and photoreceptors layer (PR). The details of the singular segmentation layer are reported in Fig. 3D. The volume and thickness of the whole retina (RT) and of each distinct retinal layer was automatically calculated by the software.

### Statistical analysis

Amplitude of the Fourier analyzed fERG 1F component, BCVA as well as thickness and volume of the retina and all retinal layers segmented by OCT software were compared between control subjects and patients with mtDNA mutations by independent two-tailed t-test. Correlation analysis between BCVA, fERG 1F amplitude and clinical parameters was performed by Pearson’s correlation. Before analysis BCVA was converted from Snellen decimals to logarithm of minimum angle of resolution (LogMAR). For the purposes of statistical analysis, the average of the data from the right and the left eye was considered, to minimize the risk of data redundancy and results overestimation. In all the analyses, a p value < 0.05 was considered significant.

## Data Availability

The datasets generated during and/or analyzed during the current study are available from the corresponding author on reasonable request.
